# Early renal structural changes and potential biomarkers in diabetic nephropathy

**DOI:** 10.3389/fphys.2022.1020443

**Published:** 2022-11-08

**Authors:** Hao Liu, Jianguo Feng, Liling Tang

**Affiliations:** ^1^ Key Laboratory of Biorheological Science and Technology, Ministry of Education, College of Bioengineering, Chongqing University, Chongqing, China; ^2^ Department of Anesthesiology, The Affiliated Hospital of Southwest Medical University; Laboratory of Anesthesiology, Southwest Medical University, Luzhou, China

**Keywords:** diabetic nephropathy, early biomarkers, glomerulus, tubule, proteomics

## Abstract

Diabetic nephropathy is one of the most serious microvascular complications of diabetes mellitus, with increasing prevalence and mortality. Currently, renal function is assessed clinically using albumin excretion rate and glomerular filtration rate. But before the appearance of micro-albumin, the glomerular structure has been severely damaged. Glomerular filtration rate based on serum creatinine is a certain underestimate of renal status. Early diagnosis of diabetic nephropathy has an important role in improving kidney function and delaying disease progression with drugs. There is an urgent need for biomarkers that can characterize the structural changes associated with the kidney. In this review, we focus on the early glomerular and tubular structural alterations, with a detailed description of the glomerular injury markers SMAD1 and Podocalyxin, and the tubular injury markers NGAL, Netrin-1, and L-FABP in the context of diabetic nephropathy. We have summarized the currently studied protein markers and performed bioprocess analysis. Also, a brief review of proteomic and scRNA-seq method in the search of diabetic nephropathy.

## Introduction

In 2021, the International Diabetes Federation counted adults (20–79 years) with diabetes mellitus (DM) up to 537 million, accounting for 10%, and it is estimated that the number of patients will reach 643 million by 2030 and 783 million by 2045 (IDF, 2021). Diabetic nephropathy (DN) is one of the most common and serious microvascular complications of DM and is associated with increased morbidity and mortality in diabetic patients (Valencia and Florez, 2017). DN accounts for 30%–50% of the incidence of end-stage renal disease, it is estimated that about 40% of patients require renal replacement therapy (Umanath and Lewis, 2018). DN puts great stress on the lives of patients, not only physically and financially but also psychologically.

Currently, the diagnosis and prognosis of DN rely on the albumin excretion rate (AER) and glomerular filtration rate (GFR). Although renal biopsy is the gold standard for the diagnosis of renal disease, it is an invasive test and is made in the presence of significant renal impairment. However, recent evidence challenges this view, Joslin’s study of the natural history of microalbuminuria suggests that the likelihood of regression from microalbuminuria to normal urinary albumin excretion is greater than the likelihood of developing significant proteinuria (Perkins et al., 2003). Likewise, in the presence of significant proteinuria, serious damage to the glomerulus has occurred, some diabetic patients with normoproteinuria have progressive renal insufficiency, called normoproteinuric diabetes (Chen et al., 2017). Therefore, urinary albumin is not sufficient and accurate as an early biomarker of DN. The use of the Schwartz formula to calculate an estimated glomerular filtration rate based on serum creatinine avoids these difficulties but underestimates the actual renal status (Salem et al., 2020).

Therefore, new biomarkers are needed to better assess the renal status of patients with DN with less influence by factors such as gender and age. Also, new biomarkers can characterize the effect of drug therapy in time to achieve the optimal dose and type of drug.

## Glomerular and tubular lesions and classification of injury

DN is accompanied by a continuous and unstoppable process of glomerular damage. It is characterized by diffuse and nodular mesangial expansion, thickening of the glomerular basement membrane, excessive accumulation of extracellular matrix, and loss of podocytes, which affect the glomerular capillaries and disrupt the structural integrity of the glomerulus (Mazzucco et al., 2002; Zheng et al., 2004). These eventually lead to an increase in proteinuria and impaired kidney function (Pourshabanan et al., 2019). Recent reports have shown that the renal tubule and interstitium play an integral role in the pathogenesis of DN and are closely associated with the progressive decline in renal function (Hills and Squires, 2011). Proximal tubular cell damage in DN includes basement membrane thickening, tubular lesions, tubular hypertrophy, tubular fibrosis (Jenkin et al., 2012).

### Degree of glomerular damage

The first obvious structural change in the kidney in response to multiple factors is the thickening of the glomerular basement membrane (GBM), even though diabetic patients have normal urinary albumin levels, as demonstrated in patients with type 1 and type 2 diabetes (T1D and T2D) (Tyagi et al., 2008; Najafian and Mauer, 2012). GBM width is a strong predictor of DN risk in patients with normoproteinuric T1D (Caramori et al., 2013). The podocyte performs an important role in maintaining the structure and filtration function of the glomerulus. Glomerular structural changes correlated with podocyte-specific injury in an animal model of diabetes mellitus (Tyagi et al., 2008). Before the appearance of proteinuria, structural and functional damage to the podocytes has occurred, such as loss of foot processes, hypertrophy, shedding, and apoptosis (Herbach et al., 2009; He et al., 2022). The reduction in GFR is associated with a reduction in glomerular filtration surface area, and these reflect early mesangial expansion (Moriya et al., 2019). Nodular mesangial sclerosis and diffuse mesangial expansion are specific lesions in DN, and more detailed studies have shown a close correlation between these two types of mesangial expansion (Kriz et al., 2017).

In 2010, Tervaert et al. (2010) classified the DN into four stages based on the type and degree of lesion in the glomerulus. The classes and lesions are briefly described below. Ⅰ) GBM thickening; Ⅱ) mesangial expansion; Ⅲ) nodular sclerosis (Kimmelstiel–Wilson lesions); Ⅳ) advanced diabetic glomerulosclerosis.

### Renal tubular injury

Some markers of proximal tubular cell injury can be detected in the urine of early diabetic patients, when there is no obvious glomerular injury, indicating that proximal tubular injury is also an early lesion and not completely secondary to glomerular injury (Chen et al., 2020). In addition to the increase in GBM thickness, tubular basement membrane (TBM) thickness is also predictive of early DN (Tyagi et al., 2008). The study confirmed that TBM thickness combined with GBM thickness provided more predictive value for patients progressing to end-stage renal disease (Zhao et al., 2021). Accompanied by inflammation, oxidative stress, and altered hemodynamics, renal tubular epithelial cells undergo cell proliferation and subsequent cell hypertrophy, cell death (Thomas, 2021; Liu et al., 2022; Uehara-Watanabe et al., 2022). In 2005, Thomas et al. described the tubular changes in early DN. Four major structural alterations of the renal tubules are highlighted, which are tubular hypertrophy and hyperplasia; tubular atrophy and dilatation; thickening of the TBM; tubular Epithelial-Mesenchymal Transition (Thomas et al., 2005).

## Glomerular and tubular injury biomarkers

The natural course of DN is characterized by lesion development and progression during a prolonged period of clinical silence, and the lesion may have developed for a long time before the AER increases and/or the GFR decreases (Parving et al., 2004). There is increasing evidence that regression of microalbuminuria is common in patients with T1D and that a significant proportion of non-albuminuric patients also develop progressive impairment of renal function (Krolewski, 2015). Therefore, the diagnosis of DN may be more accurate by looking for markers that can characterize structural alterations. We therefore reviewed the literature based on 1) the association with specific renal structural alterations in patients with DN and 2) the fact that in clinical studies, alterations in protein expression appear early in DN and have the potential to predict renal function. In this review, we present a detailed description of some of the proteins that have obtained adequate studies and are considered to have great potential to become markers of DN, but the association between other proteins and structural alterations cannot be denied.

### Markers of renal glomerular injury

The degree of mesangial expansion, one of the structural abnormalities of the glomerulus, is associated with the development of DN. In the absence of elevated blood pressure or reduced creatinine (Cre) clearance, extensive studies of glomerular structure in diabetic patients with or without microalbuminuria have found significant differences in glomerular structural changes (e. g. mesangial matrix expansion). In the Streptozotocin (STZ)-induced DN model in rats, urinary SMAD1 excretion was strongly correlated with the severity of expansion of the mesangial matrix (Matsubara et al., 2006)and can be used to predict the effect of angiotensin II type 1 receptor blocker treatment on the expansion of the mesangial matrix in DN (Mima et al., 2008). During the glomerular hyperfiltration phase, urinary SMAD1 levels were significantly elevated, indicating that mesangial expansion had occurred (Fu et al., 2013). Therefore, the role of urinary SMAD1 levels in early DN needs to be further investigated.

Podocalyxin (PCX) is a podocyte membrane protein and it is a major component of the GBM charge barrier. Glomerular filtration barrier permeability correlates with PCX integrity (Hara et al., 1994). As a marker protein of podocyte injury, podocyte injury can result in decreased levels and increased excretion of PCX in the glomerulus (Akankwasa et al., 2018). In diabetic patients, PCX protein concentration of urine supernatant is higher than the critical value in 53.8% of patients in the normal proteinuric phase, although urinary PCX levels were maintained at around 65% in the microproteinuric and massive proteinuric phases (Hara et al., 2012). Moreover, compared with the glomerular high PCX expression group, DN patients in the low expression group had a longer duration of diabetes, and the kidney survival rate in the high expression group was significantly higher than that in the low expression group (Wang et al., 2020). It indicates that PCX has some value in characterizing the onset stage of DN patients (Ye et al., 2014).

### Markers of renal tubular injury

Recent literature reports that tubular damage appears in the early stages of DN and promotes the progression of renal disease (Guo et al., 2012; Zeni et al., 2017).

In children and adolescents with T1D, NGAL fractions were detected in the extracellular vesicles of urine at higher levels than in urine from T1D patients without exosomes and in normal controls. In addition, NGAL has been present in patients without microalbuminuria or with a normal albumin-to-creatinine ratio, suggests that tubular damage occurred before the onset of classic DN symptoms (Ugarte et al., 2021). NGAL has also been shown to be a marker of early nephropathy injury in patients with T2DN (Żyłka et al., 2018; Tang et al., 2019). A lack of independent correlation between tubular injury markers and glomerular filtration rate has been reported and cannot be used to improve the management of DN, suggesting that NGAL is specific as a marker of tubular injury (Kuwabara et al., 2009; Nielsen et al., 2011). Therefore, further studies are needed for the predictive value of NGAL in early DN injury.

Netrin-1 is secreted protein highly induced after chronic and acute kidney injury. It can be detected in urine in both mice and human, and can be used as a marker for acute kidney injury (Levey et al., 2005; Reeves et al., 2008). Netrin-1 has also been reported in DN. Using a case-control study, Ay et al. showed that plasma Netrin-1 levels were significantly higher in microalbuminuric diabetic patients than in normoalbuminuric diabetic patients and controls, but there was no significant difference between normoproteinuric patients and controls (Ay et al., 2016). However, a recent study showed that Netrin-1 estimation in urine has higher accuracy than Netrin-1 estimation in serum and is a potential marker for early diagnosis of DN (Jayakumar et al., 2014). In type I diabetic animals, Netrin-1 expression was increased in proximal renal tubular epithelial cells and Netrin-1 was significantly elevated in the early phase without microalbuminuria and the late phase of all diabetic nephropathies compared to controls (White et al., 2013). However, whether Netrin-1 is affected by short-term blood glucose fluctuations requires further study (Uçaktürk et al., 2019).

Fatty acid binding protein 1 (FABP1 or L-FABP) is a small 14 kDa molecule protein expressed in the human proximal renal tubule. The circulating portion of FABP1 is filtered by the glomerulus and then reabsorbed by the proximal tubule, which explains its increased concentration in the urine when proximal tubular cells are injured (Pelsers et al., 2003). Staging of T2D patients by eGFR and urinary albumin and assessing urinary L-FABP levels in patients with different albumin levels showed that urinary L-FABP levels were significantly higher in diabetic patients with normal urinary albumin than in normal controls in the presence of renal impairment, suggesting that urinary L-FABP detects renal disease in diabetic patients earlier than urinary albumin (Thi et al., 2020). Although L-FABP levels were significantly negatively correlated with eGFR and increased with proteinuria severity, markers of tubular damage do not appear to be predictors of decreased GER in patients with T2D (Kamijo-Ikemori et al., 2011; Chou et al., 2013; Fiseha, 2015). Studies in T1D patients, suggesting that urinary L-FABP is an independent predictor of tubular damage in DN and remains useful in the early stages of DN (Panduru et al., 2013; Suh et al., 2016).

### Other protein biomarkers

**FIGURE 1:**
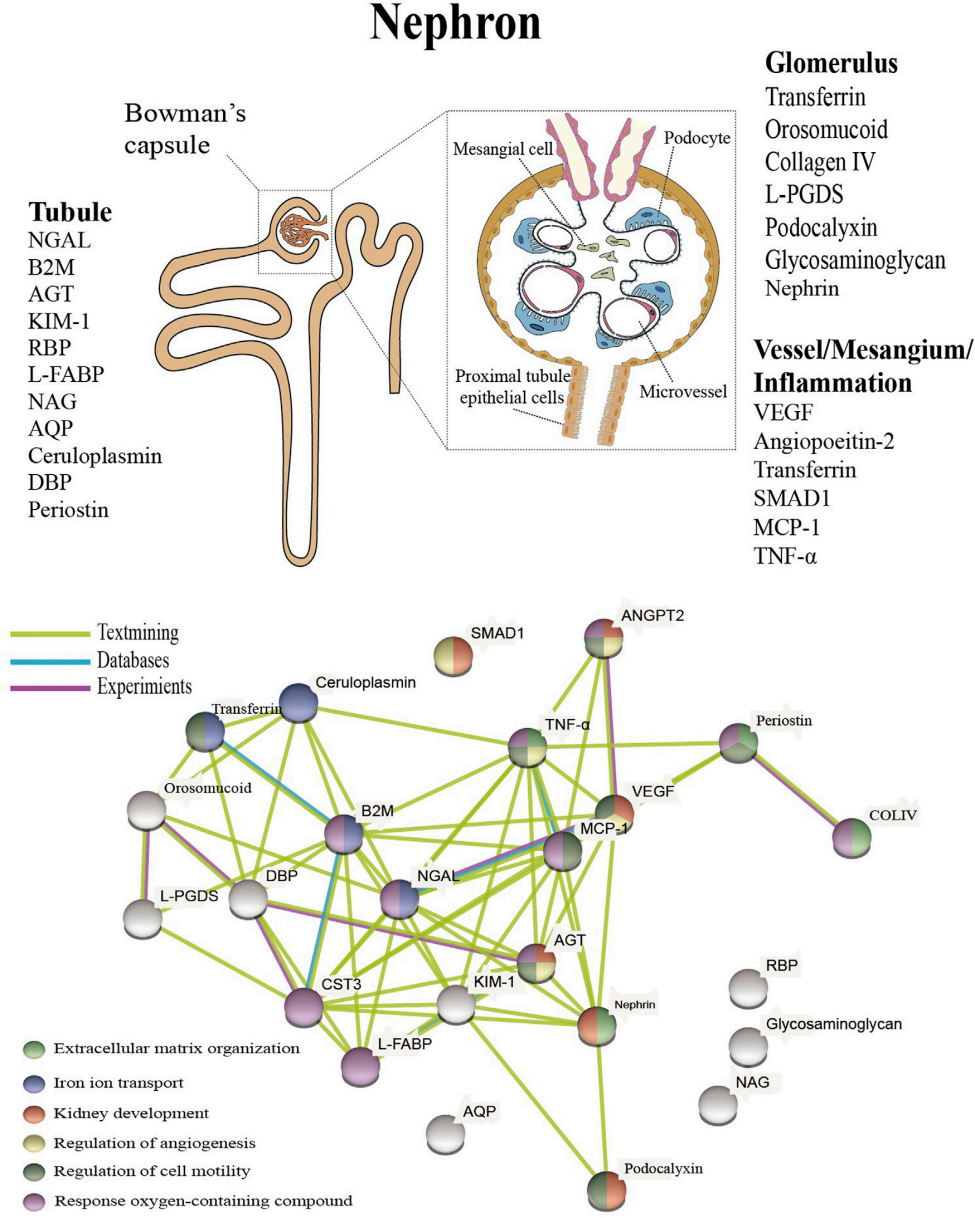
Presentation of biomarkers in the nephron (upper) and protein interaction networks and biological processes (below). Abbreviation: NGAL: Neutrophil gelatinase associated lipocalin, B2M: Beta-2-microglobulin, AGT: Angiotensinogen, KIM-1: Kidney injury molecule-1, RBP: Retinol-binding protein, L-FABP: Liver type fatty acid binding protein, COLIV: Collagen IV, NAG: N-Acetyl-B-d-glycosaminidase, ANGPT2: Angiopoietin-2, AQP: Aquaporin, DBP: Vitamin D binding protein, L-PGDS: Lipocalin-type prostaglandinD2-synthase, VEGF: Vascular endothelial growth factor, MCP-1: Monocyte chemoattractant protein-1, TNF-α: Tumour necrosis factor-alpha.

In addition to the biomarkers mentioned above, there are a large number of proteins that characterize tubular injury, glomerular filtration, mesangial dilation, vascular injury, and renal inflammation (Figure 1 upper). These proteins have been extensively studied and tested in the urine or blood of diabetic patients, and experiments have confirmed that these proteins are associated with specific structural damage and are able to characterize the development of the disease.

The pathophysiology of DN is complex and includes hemodynamic changes, oxidative stress, activation of the renin-angiotensin system, metabolic changes and various intracellular signaling (Roointan et al., 2021). Altered protein expression levels are associated with the progression of DN, and as a systemic metabolic disease, the disease may not seem to be fully characterized based on a specific protein (Zürbig et al., 2012). The above proteins are linked to specific structural damage, and bioinformatic methods can be used to better understand the linkage of proteins and the biological processes involved (Geng et al., 2019).

The protein interactions were examined using the Protein Interaction Online Analysis tool (https://cn.string-db.org/), and the results showed that these experimentally validated proteins do not exist in isolation and that there is an interrelationship between them. These proteins were also analyzed for enrichment by biological processes, and the results indicate that they are mainly enriched in extracellular matrix organization, iron ion transport, kidney development, regulation of angiogenesis, regulation of cell motility, and response oxygen-containing compounds (Figure 1 below). Oxidative stress, disturbances in lipid metabolism play a continuous role in the early stages of DN, resulting in elevated levels of kidney inflammation and increased cell death. (Wellen and Hotamisligil, 2005). Processes closely associated with the persistent early elevation of blood glucose, such as increased ion transport-related proteins transferrin and ceruloplasmin, reflect endothelial cell dysfunction and increased intra-glomerular pressure (Narita et al., 2006; Sánchez-Hidalgo et al., 2021), and upregulated expression of VEGF promotes angiogenesis (Aly et al., 2019), as well as increased collagen IV are also closely associated with the fibrotic process (Kotajima et al., 2000). A recent study showed a detailed interpretation of the progression of DN by combining proteomics and peptidomics in the urine of diabetic subjects, and the results of our analysis have similarities to this study (Van et al., 2017).

## The role of proteomics and scRNA-seq in DN research

To better understand the pathological features of DN and to search for potential biomarkers with higher specificity and accuracy, several proteomic studies have been carried out in the last years. Comparisons between diabetic subjects at different stages of renal dysfunction and controls showed differences in the expression of multiple proteins. Seven proteins were progressively upregulated with increasing proteinuria, and the transporter protein VDBP was reported for the first time in the urine of patients with DN (Rao et al., 2007). In another study, proteomic analysis identified haptoglobin as a candidate biomarker for predicting early decline in renal function, and the ratio of haptoglobin to creatinine has the ability to predict renal function in diabetic patients who have not yet exhibited significant renal disease (Bhensdadia et al., 2013). Urine has an irreplaceable role in detecting kidney status. Characterizing the urinary proteomics of patients with different stages of DN helps to understand the state of the kidney and is important for finding potential biomarkers (Papale et al., 2010). This is useful in understanding the condition of patients with DN and in finding promising treatment pathways. In addition to changes in protein levels, alterations in metabolites are also present in diabetic nephropathy, lactic acid, hippuric acid, allantoin in the urine and glutamine in the blood are the most important early diagnostic biomarkers in the pathogenesis of DN. (Roointan et al., 2021). The effects of metabolic memory on DN may be long-lasting, profoundly affecting disease development and treatment through epigenetic modifications. (Kushwaha et al., 2020).

The development of DN is a complex process, such as GBM thickening in the early stages and glomerulosclerosis and interstitial fibrosis in the later stages, involving different cell types at different stages. The application of single-cell RNA sequencing (scRNA-seq) in kidney disease has allowed us to identify cell types in tissues and provide insight into cellular damage and gene expression patterns in different stages of DN. (Latt et al., 2021). Wilson et al. (2019) analyzed a single nucleus RNA sequencing dataset from human DN and showed that the kidneys had developed mild to moderate glomerulosclerosis and interstitial fibrosis when eGFR was in the normal range. This is in addition to strong pro-angiogenic features, adaptive changes in the major cell types that promote Ki^+^ secretion, and infiltration of immune cells. Additional, scRNA-seq was used to analyze the response of DN mouse models to five common treatment regimens and found that different drugs had significantly different effects on cell types, even with combination therapy. (Wu et al., 2022). The existence of computational cell trajectory analysis methods allows to simulate the process of the kidney from a normal state to the onset of lesions, thus avoiding experimental errors. (Fu et al., 2019). scRNA-seq technology provides a more precise means for us to diagnose and treat DN.

## Conclusion

DN is one of the microvascular complications of diabetes mellitus, but the development and progression of the disease are not only caused by hyperglycemia and hypertension; genetic factors, lifestyle habits, and other coexisting diseases can all have an impact on DN. Inflammation and oxidative stress play an extremely important role in diabetes as well as in renal disease, and therefore the detection of relevant biomarkers can be used to predict, diagnose and treat DN. However, it is worth noting that some of these tests are already present during diabetes and may not be suitable as biomarkers for DN. Tubular and glomerular-related biomarkers are of immediate value in indicating kidney injury, but some of them have a lag.

The advent of new technologies has greatly helped in understanding the pathology of DN and in finding appropriate biomarkers. Combining multiple indicators to evaluate DN may have better results**.**
