# Supplementary material for: Early renal structural changes and potential biomarkers in diabetic nephropathy
Source: Front Physiol. 2022 Nov 8;13:1020443. doi: 10.3389/fphys.2022.1020443 (PMC9679365; doi:10.3389/fphys.2022.1020443)
Supplement: Supplementary file 1 [file DataSheet1.docx]

Supplementary Material

# Supplemental Table 1: Detailed information on biomarkers

| **Study** | **Study design** | **Sample** | **Source** | **Biomarker** |
| --- | --- | --- | --- | --- |
| Lacquaniti et al.,2013^[1]^ | Cross-sectional study | 50 T1D patients | Urine and Serum | NGAL |
| Aksun et al.,2017^[2]^ | Cross-sectional study | 39 with and 29 without microalbuminuria T2D patients and 32 controls | Serum | B2M |
| Siddiqui et al.,2020^[3]^ | Cross-sectional study | 185 T2D patients | Urine | Transferrin |
| Satirapoj et al.,2014^[4]^ | Cross-sectional study | 52 normoalbuminuria  52 microalbuminuria  51 macroalbuminuria T2D patients | Urine | AGT |
| Kim et al.,2004^[5]^ | Cross-sectional study | 47 non-diabetic healthy control  37 normoalbuminuric diabetic  37 microalbuminuric diabetic  33 overt proteinuric diabetic | Urine | VEGF |
| Gohda et al.,2020^[6]^ | Cross-sectional study | 602 T2D patients | Serum and Urine | KIM-1 |
| Wang et al.,2020^[7]^ | Cross-sectional study | 406 T2D patients | Urine | Orosomucoid |
| Cohen et al.,2001^[8]^ | Cross-sectional study | 109 TID or T2D patients | Serum | Collagen IV |
| Fufaa et al., 2015^[9]^ | Cross-sectional study | 224 TID patients | Urine | MCP-1 |
| Navarro et al.,2006^[10]^ | Cross-sectional study | 160 T2D patients and 32 healthy controls | Urine | TNF-α |
| Uehara et al., 2009^[11]^ | Cross-sectional study and prospective study | a、793 healthy controls and 200 patients with various forms of renal diseases.  b、666 patients with T2D patients  c、121 T2D patients | Urine | L-PGDS |
| Salem et al., 2002^[12]^ | Cross-sectional study, prospective study | 59 T1D patients, 40 matched healthy controls | Urine | RBP |
| Kondapi et al., 2021^[13]^ | Cross-sectional study | 125 T2D patients, 45 healthy controls | Urine | Nephrin |
| Hara et al., 2012^[14]^ | Cross-sectional study | 142 glomerular diseases  71 T2D patients  69 healthy controls | Urine | Podocalyxin |
| Thi et al., 2020^[15]^ | Cross-sectional study | 106 T2D patients  30 non-diabetic people | Urine | L-FABP |
| Mohammadi-Karakani et al.,2007^[16]^ | Cross-sectional study | 32 diabetes mellitus patients  25 healthy controls | Urine | NAG |
| Lu et al. 2016^[17]^ | Cross-sectional study | a、26 normal controls  25 patients with diabetes mellitus or 33 DN  b、validation cohort possessing 10 normal controls  10 patients with diabetes mellitus or 14 DN  10 patients with chronic kidney disease of unknown etiology | Urine | AQP |
| Aly et al. 2019^[18]^ | Cross-sectional study | 180 T2D patients | Urine | Angiopoietin-2 |
| Lee et al.,2015^[19]^ | follow-up | 643 T2D patients | Serum | Ceruloplasmin |
| Popławska-Kita et al.,2008^[20]^ | Cross-sectional study | 33 TID patients  53 T2D patients  30 healthy controls | Urine | Glycosaminoglycan |
| Shoukry et al.,2015^[21]^ | Cross-sectional study | 25 normoalbuminuria T2D patients  25 microalbuminuria T2D patients  25 macroalbuminuria T2D patients  25 healthy controls | Urine | DBP |
| Satirapoj et al.,2015^[22]^ | Cross-sectional study | 114 normoalbuminuria T2D patients  100 microalbuminuria T2D patients  114 macroalbuminuria T2D patients  30 healthy controls | Urine | Periostin |
| Doi et al.,2018^[23]^ | Cross-sectional study | 815 T2D patients | Urine | SMAD1 |

[1] LACQUANITI A, DONATO V, PINTAUDI B, et al. "Normoalbuminuric" diabetic nephropathy: tubular damage and NGAL [J]. Acta Diabetol, 2013, 50(6): 935-942.

[2] AKSUN S A, OZMEN D, OZMEN B, et al. Beta2-microglobulin and cystatin C in type 2 diabetes: assessment of diabetic nephropathy [J]. Exp Clin Endocrinol Diabetes, 2004, 112(4): 195-200.

[3] SIDDIQUI K, JOY S S, GEORGE T P, et al. Potential Role and Excretion Level of Urinary Transferrin, KIM-1, RBP, MCP-1 and NGAL Markers in Diabetic Nephropathy [J]. Diabetes Metab Syndr Obes, 2020, 13(5103-5111.

[4] SATIRAPOJ B, SIRITAWEESUK N, SUPASYNDH O. Urinary angiotensinogen as a potential biomarker of diabetic nephropathy [J]. Clin Kidney J, 2014, 7(4): 354-360.

[5] KIM N H, KIM K B, KIM D L, et al. Plasma and urinary vascular endothelial growth factor and diabetic nephropathy in Type 2 diabetes mellitus [J]. Diabet Med, 2004, 21(6): 545-551.

[6] GOHDA T, KAMEI N, KOSHIDA T, et al. Circulating kidney injury molecule-1 as a biomarker of renal parameters in diabetic kidney disease [J]. J Diabetes Investig, 2020, 11(2): 435-440.

[7] WANG H, BAO X, MA Y, et al. Urinary orosomucoid 1 protein to creatinine ratio as a potential biomarker for early screening of kidney impairment in type-2 diabetes patients [J]. Nephrology (Carlton), 2020, 25(9): 667-675.

[8] COHEN M P, SHEARMAN C W, LAUTENSLAGER G T. Serum type IV collagen in diabetic patients at risk for nephropathy [J]. Diabetes Care, 2001, 24(8): 1324-1327.

[9] FUFAA G D, WEIL E J, NELSON R G, et al. Urinary monocyte chemoattractant protein-1 and hepcidin and early diabetic nephropathy lesions in type 1 diabetes mellitus [J]. Nephrol Dial Transplant, 2015, 30(4): 599-606.

[10] NAVARRO J F, MORA C, MUROS M, et al. Urinary tumour necrosis factor-alpha excretion independently correlates with clinical markers of glomerular and tubulointerstitial injury in type 2 diabetic patients [J]. Nephrol Dial Transplant, 2006, 21(12): 3428-3434.

[11] UEHARA Y, MAKINO H, SEIKI K, et al. Urinary excretions of lipocalin-type prostaglandin D synthase predict renal injury in type-2 diabetes: a cross-sectional and prospective multicentre study [J]. Nephrol Dial Transplant, 2009, 24(2): 475-482.

[12] SALEM M A K, EL-HABASHY S A, SAEID O M, et al. Urinary excretion of n-acetyl-beta-D-glucosaminidase and retinol binding protein as alternative indicators of nephropathy in patients with type 1 diabetes mellitus [J]. Pediatr Diabetes, 2002, 3(1): 37-41.

[13] KONDAPI K, KUMAR N L, MOORTHY S, et al. A Study of Association of Urinary Nephrin with Albuminuria in Patients with Diabetic Nephropathy [J]. Indian J Nephrol, 2021, 31(2): 142-148.

[14] HARA M, YAMAGATA K, TOMINO Y, et al. Urinary podocalyxin is an early marker for podocyte injury in patients with diabetes: establishment of a highly sensitive ELISA to detect urinary podocalyxin [J]. Diabetologia, 2012, 55(11): 2913-2919.

[15] THI T N D, GIA B N, THI H L L, et al. Evaluation of urinary L-FABP as an early marker for diabetic nephropathy in type 2 diabetic patients [J]. J Med Biochem, 2020, 39(2): 224-230.

[16] MOHAMMADI-KARAKANI A, ASGHARZADEH-HAGHIGHI S, GHAZI-KHANSARI M, et al. Determination of urinary enzymes as a marker of early renal damage in diabetic patients [J]. J Clin Lab Anal, 2007, 21(6): 413-417.

[17] LU Y, CHEN L, ZHAO B, et al. Urine AQP5 is a potential novel biomarker of diabetic nephropathy [J]. J Diabetes Complications, 2016, 30(5): 819-825.

[18] ALY M H, ARAFAT M A, HUSSEIN O A, et al. Study of Angiopoietin-2 and vascular endothelial growth factor as markers of diabetic nephropathy onset in Egyptians diabetic patients with non-albuminuric state [J]. Diabetes Metab Syndr, 2019, 13(2): 1623-1627.

[19] LEE M J, JUNG C H, KANG Y M, et al. Serum Ceruloplasmin Level as a Predictor for the Progression of Diabetic Nephropathy in Korean Men with Type 2 Diabetes Mellitus [J]. Diabetes Metab J, 2015, 39(3): 230-239.

[20] POPŁAWSKA-KITA A, MIERZEJEWSKA-IWANOWSKA B, SZELACHOWSKA M, et al. Glycosaminoglycans urinary excretion as a marker of the early stages of diabetic nephropathy and the disease progression [J]. Diabetes Metab Res Rev, 2008, 24(4): 310-317.

[21] SHOUKRY A, BDEER S E-A, EL-SOKKARY R H. Urinary monocyte chemoattractant protein-1 and vitamin D-binding protein as biomarkers for early detection of diabetic nephropathy in type 2 diabetes mellitus [J]. Mol Cell Biochem, 2015, 408(1-2): 25-35.

[22] SATIRAPOJ B, TASSANASORN S, CHAROENPITAKCHAI M, et al. Periostin as a tissue and urinary biomarker of renal injury in type 2 diabetes mellitus [J]. PLoS One, 2015, 10(4): e0124055.

[23] DOI T, MORIYA T, FUJITA Y, et al. Urinary IgG4 and Smad1 Are Specific Biomarkers for Renal Structural and Functional Changes in Early Stages of Diabetic Nephropathy [J]. Diabetes, 2018, 67(5): 986-993.
